# Case report: Immune profiling links neutrophil and plasmablast dysregulation to microvascular damage in post-COVID-19 Multisystem Inflammatory Syndrome in Adults (MIS-A)

**DOI:** 10.3389/fimmu.2023.1125960

**Published:** 2023-02-23

**Authors:** Mark R. Gillrie, Nicole Rosin, Sarthak Sinha, Hellen Kang, Raquel Farias, Angela Nguyen, Kelsie Volek, Jordan Mah, Etienne Mahe, Marvin J. Fritzler, Bryan G. Yipp, Jeff Biernaskie

**Affiliations:** ^1^ Department of Microbiology, Immunology and Infectious Diseases, Cumming School of Medicine, University of Calgary, Calgary, AB, Canada; ^2^ Snyder Institute for Chronic Diseases, Cumming School of Medicine, University of Calgary, Calgary, AB, Canada; ^3^ Department of Medicine, Cumming School of Medicine, University of Calgary, Calgary, AB, Canada; ^4^ Department of Comparative Biology and Experimental Medicine, Faculty of Veterinary Medicine, University of Calgary, Calgary, AB, Canada; ^5^ Department of Critical Care Medicine, Cumming School of Medicine, University of Calgary, Calgary, AB, Canada; ^6^ Department of Pathology & Laboratory Medicine, Cumming School of Medicine, University of Calgary, Calgary, AB, Canada; ^7^ Department of Biochemistry and Molecular Biology, Cumming School of Medicine, University of Calgary, Calgary, AB, Canada; ^8^ Hotchkiss Brain Institute, University of Calgary, Calgary, AB, Canada; ^9^ Alberta Children’s Hospital Research Institute, University of Calgary, Calgary, AB, Canada; ^10^ Department of Surgery, University of Calgary, Calgary, AB, Canada

**Keywords:** MIS-A, COVID-19, immunophenotyping, neutrophil, plasmablast, immune dysfunction, microvascular damage, case report

## Abstract

Despite surviving a SARS-CoV-2 infection, some individuals experience an intense post-infectious Multisystem Inflammatory Syndrome (MIS) of uncertain etiology. Children with this syndrome (MIS-C) can experience a Kawasaki-like disease, but mechanisms in adults (MIS-A) are not clearly defined. Here we utilize a deep phenotyping approach to examine immunologic responses in an individual with MIS-A. Results are contextualized to healthy, convalescent, and acute COVID-19 patients. The findings reveal systemic inflammatory changes involving novel neutrophil and B-cell subsets, autoantibodies, complement, and hypercoagulability that are linked to systemic vascular dysfunction. This deep patient profiling generates new mechanistic insight into this rare clinical entity and provides potential insight into other post-infectious syndromes.

## Introduction

Despite low incidence of severe acute COVID-19 in healthy younger individuals, they are not completely spared. The most notable post-COVID-19 disease is referred to as Multisystem Inflammatory Syndrome in Children (MIS-C) characterized by an intense inflammatory disease affecting the heart, skin, and mucosal surfaces with onset weeks after primary infection (1, 2). A similar syndrome has been reported in case series of post-COVID-19 adults with marked multiorgan dysfunction including cardiac failure that spares the lungs, referred to as MIS-A (3). Five criteria have been proposed to define this condition (3) with updated criteria by the US CDC (4) and ongoing surveillance revealing hundreds of cases globally (5). Unlike the substantial body of literature on primary SARS-Cov2 infection, there is limited understanding of the mechanisms for late complications of COVID-19 in adults such as MIS-A. Little is known about the pathophysiology of MIS-A, but candidate pathways include cytokine storm, immune cell dysregulation, autoantibody production, vascular dysfunction, and immunothrombosis (6–10). The aim of the current study is to profile the immune response of a patient with MIS-A to reveal unique molecular and cellular mechanisms underlying this rare condition.

## Post-COVID-19 MIS-A case report

**Figure 1:**
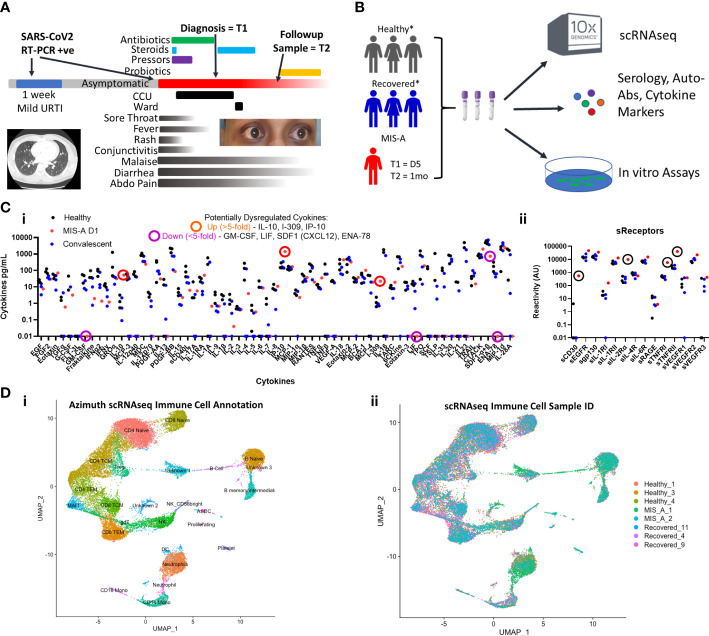
COVID-19 associated Multisystem Inflammatory Syndrome of Adults (MIS-A) is associated with critical illness, cytokine dysregulation and altered immune cell landscape. **(A)** MIS-A case outlining timeline of prior mild COVID-19 upper respiratory tract infection (URTI) and subsequent presentation of critical illness due to MIS-A with symptoms, treatments, and investigational sampling times (T=1 and T=2) highlighted. **(B)** A multi-disciplinary pre-existing COVID-19 investigational approach was utilized to investigate immune responses in this rare syndrome with relevant healthy and recovered COVID-19 controls. **(C)** Multiplex cytokine arrays (i) defined upregulated and down regulated cytokines, notably IL-10 and IP-10, and soluble cytokine receptors (ii) including sCD25 (IL2αR) as key markers of cytokine storm. **(D)** Single cell RNA seq (scRNAseq) immune cell annotation using the Azimuth database (i) with identification of cellular source (ii) identifying increased B cell and neutrophil populations in MIS-A.

A 38-year-old unvaccinated South Asian male presented to a tertiary care hospital complaining of a 4-day history of fever, abdominal pain, and diarrhea. The patient was previously healthy and had no known relevant family or psychosocial history. On exam he was noted to have a macular blanching skin rash on his torso and proximal extremities, bilateral non-purulent conjunctivitis, sore throat, and prominent non-exertional chest pressure associated with progressive shortness of breath (**Figure 1A**). In the emergency department he was febrile, hypotensive due to cardiogenic shock, and started on vasopressors to maintain systolic blood pressure and was eventually transferred to the Cardiac Care Unit (CCU) for cardiac support (**Figure 1A**, **Supplementary Table 1**).

Twenty-five days prior to presentation, the patient had several days of cough and sore throat without any other respiratory symptoms. Both the patient and household family members tested positive for SARS-CoV2 by RT-PCR of nasopharyngeal swabs. These symptoms had completely resolved for more than two weeks prior to the most recent presentation.

Initial laboratory investigations on admission revealed elevated inflammatory, renal, hepatic, and coagulation dysfunction markers (**Supplementary Table 1**). An echocardiogram showed severe biventricular heart failure. An initial chest Computed Tomography (CT) scan using a pulmonary embolism protocol was negative with remarkably normal lungs (**Figure 1A**). A SARS-CoV2 NP RT-PCR test was low positive (Ct-value >30) with the remainder of the respiratory viral pathogen panel negative. SARS-CoV2 serology to spike RBD and N proteins was strongly positive. The patient was presumptively diagnosed with myocarditis and potential acute coronary syndrome while being investigated for sepsis. He was started on aspirin, enoxaparin, and broad-spectrum antibiotics in consultation with cardiology and infectious diseases clinical teams.

While in CCU, he was slowly weaned off vasopressors but developed worsening leukocytosis with neutrophilia, renal failure with evidence of a procoagulant state (increased D-Dimer), further elevated inflammatory (C-reactive protein) and cardiac dysfunction (NT-proBNP and troponin) markers. He had continued diarrhea and severe abdominal pain leading to escalation of his antimicrobial therapy to meropenem. The rest of his infectious and autoimmune disease workups were negative aside from evidence of complement activation (low C3/C4 levels) (**Supplementary Table 1**). CT imaging of his head, neck, chest, and abdomen were normal. Eventually while still in CCU he had an indium white blood scan that did not show any active infection or inflammation (Supplementary **Figure 1**).

Given the constellation of extra-pulmonary systems involvement without respiratory disease, recent resolved SARS-CoV2 infection, and positive serology, he was diagnosed with COVID-19-associated MIS-A meeting all published criteria for the condition (3, 4). Antibiotics were stopped and 6mg of oral dexamethasone daily for 5 days was started. A repeat echocardiogram prior to discharge showed normal right and left ventricular function which was corroborated by cardiac MRI showing no signs of myocarditis (Supplementary **Figure 1**). The patient was discharged after 15 days, 10 of which were in the CCU.

## Materials and methods

See supplemental Materials and Methods.

## Results

### MIS-A deep immune profiling

To better contextualize our findings, we compared both acute and convalescent blood samples from our MIS-A patient to three age-matched healthy and convalescent COVID-19 controls at similar time frames post infection (**Supplementary Table 2** and **Figure 1B**, see Supplemental Materials and Methods) using a published precision medicine approach (11).

We first set out to establish the presence of ‘cytokine storm’ mediators reflecting a state of systemic inflammation. Analysis of >75 cytokines and soluble receptors highlighted 5-fold elevated levels of sCD25R, CTACK, I-309, IL-10 and IP-10 (CXCL10) in acute MIS-A as compared to healthy and convalescent controls (**Figure 1C**), the latter of which has been implicated in MIS-C (7). Our results contrast those in severe COVID-19 where IL-6, MCP-2 (CCL2), G-/GM-CSF, and IFN-γ are often high (12). Additionally, we found elevated circulating calprotectin (S100A8/9, MIS-A = 298 picogram/mL, all controls <14pg/mL) an ‘alarmin’ associated with neutrophils that has been found elevated in severe COVID-19 (13) and MIS-C patients (6).

### scRNAseq reveals emergence of neutrophil and B cell subpopulations during MIS-A

We next utilized single-cell RNA sequencing (scRNAseq) to further delineate circulating immune cell alterations in MIS-A compared to healthy and COVID-19 convalescent controls (11). This revealed notable decreases in total numbers of T, NK, and mononuclear cells but increases in neutrophil and B-cell populations during acute MIS-A (**Figures 1D**). Global transcriptional changes during admission demonstrated differences in B cell, naïve CD4 T cells, and neutrophil populations which, aside from some sustained abnormalities in neutrophils, were largely resolved by day 30 in MIS-A (**Supplementary Figure 3**).

**Figure 2:**
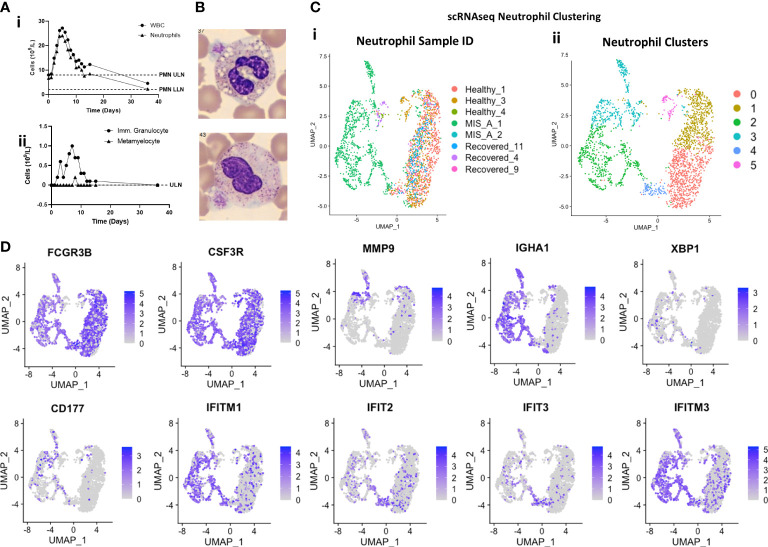
Immature, B-cell like, and ‘IFN’ neutrophil subpopulations emerge in MIS-A. **(A)** Total peripheral blood neutrophil (PMN) counts (i) transiently increase in MIS-A with concurrent increases in immature neutrophil populations (ii). **(B)** Morphologic assessment demonstrates ‘toxic’ changes in neutrophils with increased vacuolization and cytoplasmic irregularities (i) plus immature ‘band’ forms (ii). **(C)** scRNAseq analysis of all neutrophils identifies increases in neutrophils in MIS-A (i) with increases seen in three (2-4) of six (0-5) neutrophil subpopulations (ii). **(D)** Neutrophil mRNA expression by scRNAseq of classical neutrophil markers (FCGR3B, CSF3R), immature neutrophils (MMP9), B cells (IGHA1, XBP1), neutrophil activation (CD177), and interferon (IFN) stimulated genes (IFITM1 and 3, IFIT2-3).

Neutrophil counts were strikingly elevated in MIS-A with increased immature neutrophil populations in clinical blood counts (**Figure 2A**). Consistent with this, peripheral blood smears demonstrated a large percentage of neutrophil progenitor cells including ‘band’ forms with features of metamyelocytes, and neutrophils with ‘toxic changes’ suggestive of activation and phagocytosis (**Figures 2A, B** and **S4**) not typically seen healthy individuals. scRNAseq analysis of total neutrophil populations revealed elevated markers of immaturity (MMP9), B cells (e.g. MZB1, Immunoglobulins, XBP1) and inflammation (CD177, CST7) (**Figures 2D** and **S5**). Unsupervised clustering of neutrophils from all scRNAseq samples identified six (‘0-5’) neutrophil subpopulations expressing common markers FCGR3B and CSF3R (**Figures 2C–D**). Clusters 0,1,5 were shared by all clinical groups but predominantly represented by healthy and convalescent patients (**Figure 2C**). In contrast, cluster 2-4 neutrophils were exclusive to MIS-A with 2 and 3 co-expressing immature neutrophil (MMP9), activation (CD177), and B cell markers (IGHA1 and XBP1) while cluster 4 had greater expression of IFN stimulated genes (ISGs) including IFIT2 and IFIT3 yet did not map to our previously identified PMN populations in severe COVID-19 (**Figures 2D** and **S5, 6**) (11).

**Figure 3:**
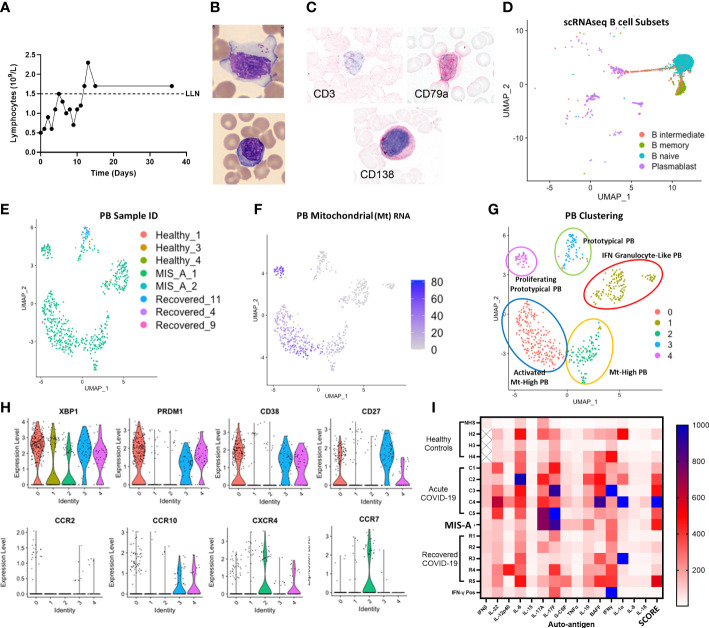
MIS-A is associated with lymphopenia but increased atypical lymphocytes, Plasmablast **(PB)** B cells and autoantibody production. **(A)** Lymphopenia in acute MIS-A on peripheral blood counts. **(B)** Emergence of atypical lymphocytes with increased cytoplasmic content **(i)** and less frequent observed but plasma cells (ii). **(C)** Staining of atypical lymphocytes demonstrate absence of T cell marker CD3 but presence of plasma/plasmablast markers CD79a and CD138 (red). **(D)** scRNAseq B-cell clustering using Azimuth reference database showing unknown but unique populations of plasmablasts (PB, purple). **(E)** Unsupervised clustering of scRNAseq plasmablasts shows increases in unique subpopulations with MIS-A. **(F)** Mitochrondrial (Mt) RNA content of PBs differentiates novel PB populations. **(G)** Proposed nomenclature for PB subsets based on top differentially expressed genes in PB subsets by scRNAseq. **(H)** Key PB genes (XBP1, PRDM1, CD38, CD27) and chemokine receptors (CCR2,7,10 and CXCR4) to differentiate PB subsets expressing Igs (see supplementary Supplementary Figure 9) and Mt RNA (panel F) in MIS-A and controls. **(I)** Serum auto-antibody reactivity to human cytokines from pooled normal healthy serum (NHS) and healthy (H2-4), acute severe COVID-19 (C1-5), MIS-A D1 and recovered COVID-19 (R1-6) patients versus known IFN-γ positive patient expressed as arbitrary units from assay. SCORE indicates an adjusted rank sum across each target to generate an autoimmunity ‘score’ for each patient. See methods for details.

We also found decreased total lymphocyte numbers during acute MIS-A but morphologically increased large granular lymphocytes (also known as ‘activated’ or ‘atypical’ lymphocytes) (**Figures 3A, B** and **S7**) which are rare in healthy controls. These atypical lymphocytes stained largely negative for T/NKT cell marker CD3 but positive for plasma/plasmablast (PB) markers CD79a and SDC1/CD138 (**Figure 3C**). Using scRNAseq we found typical B-cell populations in all groups but unique PB populations in MIS-A (**Figures 3D** and **S8–10**). PB subpopulations have been linked with rapid expansion, short survival (<1-2 weeks), and potentially damaging autoantibody production in human diseases including COVID-19 (14–16). Initially we noted three unique PB subpopulations by scRNAseq two of which were unique to MIS-A (Supplementary Figure 8). However, during scRNAseq quality control steps, we noticed that MIS-A uniquely had higher RNA and mitochondrial (MT) reads of >15%, typically considered ‘dead’ cells, and these MT^hi^ cells were >75% PBs (**Figures 3E** and **S9**), something we had not seen in our previous studies on acute critical COVID-19 (11). Unsupervised clustering including these MThi cells identified five PB clusters (‘0-5’) all of which were CD19, CD20 (MS4A1), and IgD-negative (data not shown) but positive for other immunoglobulins and B-cell markers. Four PB clusters were exclusive to MIS-A and three represented MT^hi^ populations (**Figures 3E, F** and **S9, 10**). These MIS-A-specific Ig-expressing PB subpopulations could be differentially identified by MT RNA content, proliferation (MKI67), and chemokine receptor (CXCR4/CCR7/CCR10) expression (**Figures 3E, H** and **S9, 10**).

### MIS-A is linked to autoreactive antibody production, microvascular damage, and coagulation

**Figure 4:**
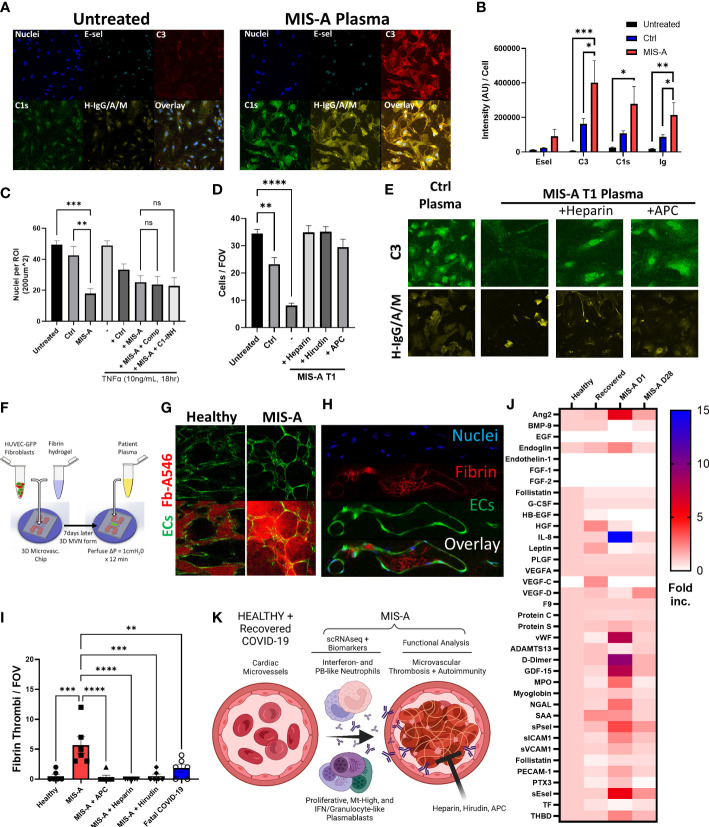
Microvascular dysfunction in MIS-A. **(A)** Primary human cardiac microvascular endothelial cells (HCMEC) untreated or treated for 30 minutes with MIS-A plasma and stained for nuclei, endothelial inflammatory markers (E-sel), complement (C3, C1s) and human IgG/A/M. **(B)** Quantitation of HCMEC staining from panel A including additional controls. **(C)** Quantitation of total endothelial cells (nuclei) as in panel A from control or MIS-A treated HCMEC pre-incubated with TNF-α (1ng/mL 18 hours) and or complement inhibitors, Compostatin (Comp) or C1 esterase inhibitor (C1-INH), as indicated. **(D)** Quantitation of total endothelial cells (nuclei) from control or MIS-A treated HCMEC pre-incubated with anti-coagulants heparin, hirudin, and activated protein C as indicated. **(E)** Representative images for C3 and H-IgG/A/M staining as in **(D–F)** 3D human microvascular model containing EGFP expressing human umbilical vein endothelial cells, normal human fibroblasts, and perfused with patient plasma containing Alexa 546-labeled fibrinogen. **(G)** Intra- and extra-vascular fibrin accumulation in healthy or MIS-A following 15-minute perfusion of patient plasma spiked with fibrinogen-Alexa546 (red) through microvascular networks (green). **(H)** Representative images of microvascular fibrinogen Alexa-546 accumulation in microvessels in response to MIS-A plasma for 15 minutes. **(I)** Quantitation fibrin accumulation as in B using patient plasma plus or minus addition of activated protein C (APC), heparin, or hirudin in comparison to healthy or fatal COVID-19 plasma. **(J)** Cytokine arrays from healthy controls (n=5) and acute *vs* convalescent MIS-A plasma (n=1 each) showing elevation in multiple vascular endothelial damage markers (Ang2, sEsel, THBD), neutrophil activation markers (MPO, NGAL) and pro-coagulants (D-Dimer, VWF, sPsel) as fold increase from healthy. **(K)** Summary of findings including emergence of plasmablast subsets, auto-antibody production, neutrophil activation, and thrombosis culminating in vascular damage in acute MIS-A. ns = nonsignificant, **p<0.01, ***p<0.001 by one-way ANOVA with *post-hoc* Tukey’s test. *p < 0.05 and ****p < 0.0001.

To determine potential pathogenic consequences of PB dysfunction, we investigated SARS-CoV2 and autoreactive antibody production. This indicated elevated total IgA, SARS-CoV2 antibody responses, and broad autoreactivity to multiple cytokines and human cardiac microvascular endothelial cells (HCMEC) in MIS-A (**Figures 3I** and **4A**). We found that in addition to autoantibody binding specifically to cardiac but not lung endothelium (**Supplementary Figure 12**), MIS-A plasma also resulted in classical complement deposition and HCMEC disruption in vitro (**Figures 4A–D**). However, only inhibition of coagulation using hirudin, heparin, or activated protein C but not blockade of complement activity prevented endothelial disruption (**Figures 4C–E**). Using a more physiologic 3D microvascular assay (17, 18), we found acute MIS-A more so than severe COVID-19 plasma caused microvascular leak and coagulation that was also inhibited by hirudin, heparin, or activated protein C (**Figures 4F–I**, **S13, 14**, and **Movie S1**). Exploration of plasma markers of vascular dysfunction confirmed evidence of endothelial damage (Endoglin, PECAM-1, Ang-2, sEsel, sICAM1), neutrophil activation (MPO, GDF-15), and thrombosis (vWF, D-Dimer, sPsel) in MIS-A plasma but not healthy or convalescent controls (**Figure 4J**).

## Discussion

Here we provide an in-depth immunologic analysis of an uncommon but severe late complication of COVID-19 predominantly in a young healthy male of non-Caucasian descent. Greater than 200 cases of MIS-A have now been reported with our patient being a prototypical severe case accompanied by elevated inflammatory markers (5). In contrast to MIS-C in children, MIS-A is often misdiagnosed or missed altogether in adults, which has led to difficulties in treating and examining the pathophysiology of the disease (19). Despite the syndromes striking clinical presentation, only a few case reports of MIS-A have investigated potential pathogenic features demonstrating evidence of cytokine dysregulation (‘cytokine storm’), and vascular inflammation including microvascular neutrophil accumulation and complement deposition within the heart consistent with a vasculitis (1, 20, 21). In potentially related pediatric MIS-C, cytokine dysregulation, anti-endothelial autoantibody production (6, 10, 22), and inborn errors of OAS and RNAse- L antiviral signaling (10) are known to occur in a subset of patients but the interplay between these proposed pathways, terminal effectors, and relevance to MIS-A are unclear.

During our analysis, we found evidence of multiple striking immune cell alterations in neutrophil and B cell subpopulations. To our knowledge, no other scRNAseq analysis of MIS-A exists. Parallel studies in MIS-C exist and identify plasmablast and neutrophil dysfunction (6) with others showing acute NK and T cell alterations (22), or compare responses of convalescent samples stimulated with potentially unrelated agonists (10). Our results are difficult to compare to MIS-C data given other studies using scRNAseq have used PBMCs that underrepresent neutrophil responses (11) and are also confounded by potential differences between pediatric and adult immune systems (23).

In MIS-A, we specifically identified increased immature neutrophil populations that appear to overlap somewhat with those in other disease states (11, 14, 15, 24). However, none of the overlap with previously reported neutrophil subsets was strong suggesting that although loose connections between neutrophil populations are present in different diseases, there remains significant plasticity in human neutrophil phenotypes in different disease states that is not easily reconciled even by scRNAseq. The most unexpected neutrophil difference in our study was the emergence of B-cell gene expression in neutrophils which has been seen in severe COVID-19 (14, 15). In conjunction with known rapid induction of and clearance of PBs including unique populations identified here, it is tempting to speculate that neutrophil subsets may be activating or clearing PBs in MIS-A and possibly other conditions. Confirming the presence and mechanisms of neutrophil-PB interactions in MIS-A may identify novel therapeutic targets for this and related conditions.

## Conclusions

The pathological importance of potential neutrophil and B-cell dysregulation in MIS-A to date has remained unclear. The possibly overlapping syndrome of MIS-C suggests autoantibodies and neutrophil activation lead to damage of the vasculature (6). However, a terminal pathway downstream of neutrophil and B-cell activation driving vascular dysfunction in our hands appears to be microvascular coagulation (**Figure 4K**). This concept is supported by derangements in multiple coagulation markers from MIS-A plasma, most notably the common finding of elevated fibrin degradation products (D-dimer) (5). The process of coagulation in the context of inflammation and neutrophil activation is often referred to as immunothrombosis (25). It may be that MIS-A represents a complication from a delayed inflammatory phase of severe acute COVID-19 where IgA antibodies have been shown to cause neutrophil activation via release of neutrophil extracellular traps (NETs) (26). Our 2D and 3D in vitro assays suggest that anticoagulation using direct inhibitors of the coagulation cascade including heparin, hirudin, or activated protein C (APC) may be a way to prevent vascular dysfunction. APC is particularly intriguing because it also has potent vascular protective functions through the endothelial protein C receptor (EPCR), independent of anti-coagulant activity (27).

## Limitations and future directions

Our study represents a single case of this rare condition and is therefore limited in its broad applicability but does provide a detailed roadmap for ongoing investigations of MIS-A and similar post-infectious conditions. These other conditions include MIS-C, Kawasaki disease, and even Long COVID-19, the latter of which our patient would have later fulfilled criteria for based on approximately six months of significant lingering fatigue, abdominal, and neurocognitive (‘brain fog’) symptoms. Our results demonstrate the presence of potentially novel human subpopulations of neutrophil and B cells that require further exploration in other cases of MIS-A and inflammatory conditions. Importantly, although we define differences in circulating numbers, morphologic features, and gene expression in these cells, all potential identifications by scRNAseq are proposed and do not imply functional properties. Utilization of cell surface markers are required to select and interrogate functional properties of these subpopulations. Important functional readouts to explore in subpopulations include PB cytokine and immunoglobulin production and neutrophil ROS, NET release, and degranulation. Ascertaining microvascular function in the heart and other organs of these patients would also be of benefit (28). Finally, clinical trials of proposed interventions, including anti-coagulation or APC, are desperately needed for this and other post-infectious syndromes.

## Data availability statement

The datasets presented in this study can be found in online repositories. The names of the repository/repositories and accession number(s) can be found below: https://www.ncbi.nlm.nih.gov/geo/, GSE171052, https://www.ncbi.nlm.nih.gov/geo/, GSE157789.

## Ethics statement

The studies involving human participants were reviewed and approved by Research Ethics Board, University of Calgary. The patients/participants provided their written informed consent to participate in this study. Written informed consent was obtained from the individual(s) for the publication of any potentially identifiable images or data included in this article.

## Author contributions

MG, NR, MF, BY and JB designed the study. MG, NR, HK, RF, AN, KV, MF, and EM performed the experiments and analyzed data. NR and SS performed scRNAseq and bioinformatic analysis. MG, NR, MF, and BY wrote the manuscript. All authors contributed to the article and approved the submitted version.
