# Supplementary material for: Case report: Immune profiling links neutrophil and plasmablast dysregulation to microvascular damage in post-COVID-19 Multisystem Inflammatory Syndrome in Adults (MIS-A)
Source: Front Immunol. 2023 Feb 23;14:1125960. doi: 10.3389/fimmu.2023.1125960 (PMC9995372; doi:10.3389/fimmu.2023.1125960)
Supplement: Supplementary file 1 [file DataSheet_1.pdf]

Supplementary Materials and Methods:

Clinical Cases and Ethics. MIS-A case was enrolled from cardiac care unit (CCU) upon provisional diagnosis of MIS-A on D5 of admission (T1) when samples were collected for scRNAseq, biomarker and functional assays using recently published protocol (1). Similarly serial blood smears were prospectively collected as part of a related study (2). Thirty days after initial sample (T1), a second series of blood samples were collected (T2). Healthy patients were self-identified and enrolled via an approved public noticed. Ethics approval (REB20-0481and REB20-1164) for all studies were obtained from the University of Calgary Conjoint Health Research Ethics Board (CHREB). Informed consent was obtained and documented for all patients.

Cytokine receptor and soluble cytokine arrays. Cytokines, chemokines and soluble cytokine receptors were quantitated on multiplex bead-based arrays that included a 65 MILLIPLEX cytokine/chemokine (6Ckine, BCA-1, CTACK, EGF, ENA-78, Eotaxin, Eotaxin-2, Eotaxin-3, FGF-2, Flt-3L, Fractalkine, G-CSF, GM-CSF, GRO, I-309, IFN-α2, IFN-γ, IL-1α, IL-1β, IL-1ra, IL-2, IL-3, IL-4, IL-5, IL-6, IL-7, IL-8, IL-9, IL-10, IL-12 (p40), IL-12 (p70), IL-13, IL-15, IL-16, IL-17A, IL-18, IL-20, IL-21, IL-23, IL-28a, IL-33, IP-10, LIF, MCP-1, MCP-2, MCP-3, MCP-4, MDC, MIP-1α, MIP-1β, MIP-1d, PDGF-AA, PDGF-AB/BB, RANTES, SDF-1α, SDF-1β, sCD40L, SCF, TARC, TGFa, TNFa, TNFb, TPO, TRAIL, TSLP, VEGF) and a 14 MILLIPLEX soluble cytokine (sCD30, sEGFR, sgp130, sIL-1RI, sIL-1RII, sIL-2Ra, sIL-4R, sIL-6R, sRAGE, sTNF RI, sTNF RII, sVEGF R1, sVEGF R2 and sVEGF R3) (Millipore Sigma) on a Luminex 200 luminometer. EDTA plasma samples were collected from each patient by venipuncture after a standard operating protocol and stored at −80 °C until tested. Each run included a full range of calibrators. Calprotectin was determined by ELISA (Inova Diagnostics, San Diego, CA) and in house validation showed all healthy and recovered patients <14.0 pg/mL, with larger internal validation studies showing <50pg/mL considered negative, 50-120pg/mL intermediate, and >120pg/mL considered high.

Serology and auto-antibody assays. Serology and auto-antibody panels including xMAP SARS-CoV2 IgG (Luminex Corp., Austin, TX: FDA, EUA)) testing antibodies to S1 spike, receptor binding domain (RBD) of S1 and Nucleoprotein, with a normal reference range of 0 – 700 median fluorescence intensity (MFI)); COVID ELISA IgA, G Ratio (Euroimmun AG, Luebeck, Germany: FDA EUA)), anti-cytokine (Luminex Corp.) , normal reference range 0-500 MFI), were all used according to the manufacturers protocols with fresh frozen EDTA plasma as described above. Auto-antibody scores were generated by ranking autoreactivity of each cytokine target (auto-antigen) amongst the total patient population then summing the ranks for each patient to generate a cumulative autoimmunity score which was expressed as 1.04^score to generate values that would fully utilize the color scale of graph in Figure 6C.

Anti-nuclear antibodies (ANA) were detected by an indirect immunofluorescence assay (IFA) on HEp-2 substrates (NOVA Lite HEp-2, Inova Diagnostics Inc. San Diego, CA, USA) at a dilution of 1:80 and read on an automated instrument (NOVA View, Inova Diagnostics) which interpolates fluorescence intensity to an end point titer (3). IFA staining patterns were classified according to the International Consensus on Autoantibody Patterns (ICAP: https://anapatterns.org/index.php) (4). If multiple patterns were noted in individual sera, they were recorded as separate ICAP patterns and accompanying titers.

All samples were also tested for autoantibodies associated with a spectrum of autoantibodies related to systemic autoimmune diseases (SARA) as detected by a commercially available addressable laser bead immunoassay (ALBIA: Connective 13; TheraDiag, Paris, France) which included DNA, histones, Sm/U2-U6 RNP, U1-RNP, SSA/Ro60, SSB/La, Ro52/TRIM21, ribosomal P, Ku, PCNA, topoisomerase I and CENP-B.

CellaVision Hematopathology. Peripheral smears were digitally scanned using the CellaVision DC-1 digital morphology analyzer (CellaVision AB, Lund, Sweden), with settings for a 500 cell differential count as previously described (2). Occasional specimens with fewer than 500 detectable cells were not specifically excluded. Automated cell identification and pre-classification was then performed using the CellaVision Peripheral Blood Application Software, followed by manual verification. This latter step was undertaken independently by local hematopathologists, with validity assessed by Bland-Altman analysis of independent reviews by external hematopathologists using the CellaVision Remote Review 8 Software.

Immunohistochemistry. Cells of interest identified in the peripheral smears were marked-off with a tungsten-carbide pen. Peripheral smears were then destained using a series of ethanol washes. Automated immunohistochemistry was then performed using the Leica Bond-III automated IHC system. Monoclonal CD3 (clone LN10), CD20 (clone L26), CD79a (11D10) and CD138 (clone MI15) mouse anti-human primary antibodies were used, with polymer-based Fast Red detection.

Leukocyte and lymphocyte preparation. Leukocytes and lymphocytes were isolated as previously published (1). In brief, for lymphocyte preparation, whole blood was spun (15min, 3000rpm, Room Temperature [RT]) and plasma was removed. Isolation Cocktail and Rapid Spheres (Easy Sep^{TM} Direct Human Total Lymphocytes Isolation Kit: 19655, StemCell Technologies) were added to remaining whole blood. Samples were mixed and incubated for 5min incubation at RT, the sample volumes topped up to 5mL with D-PBS+2%FBS + 1mM EDTA. The diluted sample was incubated in the magnet for 5min, at RT. This last step was repeated twice before cell resuspension in 5mL of PBS+0.04% BSA. After 2 washes 7500 lymphocytes were resuspended in 25µL of PBS+0.04% BSA.

For leukocytes preparation, whole blood was collected in heparin containing vacutubes and mixed with 0.5M EDTA with PBS+2% FBS and EasySep RBC Depletion spheres (EasySep^{TM} RBC Depletion Reagent: 18170, Stem Cell Technologies). After 5 min of magnet incubation, at RT, tubes were inverted and poured into a new tube and RBC depletion was repeated. After 2 washes, cells were resuspended in 25µL of PBS+0.04% BSA.

Single-cell RNA-Seq library construction, alignment, and quality control. A total of 15,000 single cells (containing an equal proportion of leukocytes and lymphocytes) were loaded for partitioning using 10X Genomics NextGEM Gel Bead emulsions. All samples were processed as per manufacturer's protocol (with both PCR amplification steps run 12X). Quality control and pre-loading cDNA quantification was performed using TapeStation D1000 ScreenTape assay. Sequencing was performed using Illumina NovaSeq S2 and SP 100 cycle dual lane flow cells over multiple rounds to ensure each sample received approximately 32,000 reads per cell. Sequencing reads were aligned using CellRanger 3.1.0 pipeline (5) to the standard pre-built GRCh38 reference genome. Samples that passed alignment QC were aggregated into single datasets using CellRanger aggr with between-sample normalization to ensure each sample received an equal number of

mapped reads per cell. Aggregated healthy (n = 3) and recovered COVID-19 (n = 3) samples recovered 30,514 cells that were sequenced to 17,157 post-normalization reads per cell.

Single-cell RNA-Seq computational analyses and workflows. Filtered feature-barcode HDF5 matrices from aggregated datasets were imported into the R package Seurat v.3.9 for normalization, scaling, integration, multi-modal reference mapping, louvain clustering, dimensionality reduction, differential expression analysis, and visualization (6). Briefly, cells with abnormal transcriptional complexity (fewer than 500 UMIs, greater than 25,000 UMIs, or greater than 15% of mitochondrial reads) were considered artifacts and were removed from subsequent analysis. Cell identity was classified by mapping single cell profiles to the recently published PBMC single-cell joint RNA/CITE-Seq multi-omic AZIMUTH reference (7). Since no published reference automates granulocyte annotations, neutrophil clusters were manually annotated by querying known markers (i.e. CSF3R, S100A8, S100A9, MMP8, MMP9, ELANE, MPO) (8). A cell state-specific 'perturbation score' was calculated to reflect the magnitude of response elicited by factoring in number and cumulative FC of consensus DEGs. Perturbation scores were visualized using Nebulosa-generated density plots (9). Cells with high mitochondrial read were subsetted to enable further characterization of these cells, as there were high mitochondrial reads specifically in the first MIS-A sample.

Human cardiac and lung microvascular endothelial assays. HCMEC or HLMEC (Passage 3-5, Lonza Biosciences Inc.) cultured in 96-well plates until 24 hours post-confluent. Endothelial cells were incubated for 30 minutes at 37 degrees with EDTA plasma reconstituted 1:20 with endothelial media (Vasculife MV, Lifeline Tech). Experiments with HLMEC additionally included 7.5µg/mL fibrinogen-Alexa546 (Invitrogen) in EDTA plasma. Supernatants were immediately aspirated and cells fixed with 1% PFA at room temperature and then blocked and permeabilized for 1 hour at room temperature using PBS with 2.5% BSA and 0.3% TX-100. Blocked monolayers were then stained sequentially for highly cross-absorbed biotinylated goat anti-human IgG/IgA/IgM antibody (Jackson Labs) followed by washing, and then incubation with E-sel (mouse mAb, 1:100, Cat 555648, BD Biosciences), C1s (sheep pAb, 1:500, Cat AF2060, R&D Systems), and C3 (rabbit pAb, 1:500, Cat 21337-1, Proteintech). Secondary antibodies were all used at 1:500 and include goat anti-mouse BV510 (Biolegend), goat anti-rabbit Alexa 555, donkey anti-sheep-Alexa488, and streptavidin-Alexa 647 with 1uM Hoescht DNA dye added to all mixtures for nuclear staining (latter four reagents from Invitrogen). After washing, cells were immediately imaged on a Leica Sp8 scanning confocal microscope using a 20x NA 0.95 water objective and images quantitated offline using Leica LAS-X and ImageJ software.

3D human microvascular assays. Human umbilical vein endothelial cells (HUVEC, 10-donor pooled, Lonza) were labeled with EGFP via lentiviral transfection and used at P5-7 using a previously published protocol (Nature Methods). In brief, HUVEC-GFP were mixed with NHLF (Lonza) to a final ratio of 10x10^6/ml HUVEC to 2x10^6/mL NHLF with 2.5mg/mL final human plasmin-depleted fibrinogen (EMD-Millipore) activated by 4U/mL thrombin (Sigma) to encapsulate cells within a fibrin hydrogel. After 7 days of daily media changes, media was removed and cryopreserved human plasma diluted 1:20 with HBSS and spiked with 7.5µg/mL Fibrinogen-Alexa546 (Invitrogen) was perfused through microvessels for 10 minutes. Fibrinogen deposition was imaged in real-time using an Sp8 resonant scanning microscope at 1-2 minute intervals.

Statistical Analysis. All data was analyzed using R (scRNAseq) as described in computational analysis section, otherwise Graphpad PRISM v9.2 using one or two-way ANOVA with post-hoc Tukey's multiple comparison test was used for all other data.

Supplementary References:

1. Sinha S, Rosin NL, Arora R, Labit E, Jaffer A, Cao L, et al. Dexamethasone modulates immature neutrophils and interferon programming in severe COVID-19. Nat Med. 2022;28:201–11.
2. Kubik T, Hou M, Traverse T, Lareau M, Jenei V, Oberding L, et al. Risk Assessment of Hospitalized Severe Acute Respiratory Syndrome Coronavirus 2 (SARS-CoV-2)–Infected Patients Using Laboratory Data and Immune Cell Morphologic Assessment. Arch Pathol Lab Med [Internet]. 2021 Sep 20;146(1):26–33. Available from: https://doi.org/10.5858/arpa.2021-0368-SA
3. Copple SS, Jaskowski TD, Giles R, Hill HR. Interpretation of ANA indirect immunofluorescence test outside the darkroom using NOVA view compared to manual microscopy. J Immunol Res [Internet]. 2014/02/24. 2014;2014:149316. Available from: https://pubmed.ncbi.nlm.nih.gov/24741573
4. Chan EKL, Damoiseaux J, Carballo OG, Conrad K, de Melo Cruvinel W, Francescantonio PLC, et al. Report of the First International Consensus on Standardized Nomenclature of Antinuclear Antibody HEp-2 Cell Patterns 2014–2015. Front Immunol [Internet]. 2015;6:412. Available from: https://www.frontiersin.org/article/10.3389/fimmu.2015.00412
5. Zheng GXY, Terry JM, Belgrader P, Ryvkin P, Bent ZW, Wilson R, et al. Massively parallel digital transcriptional profiling of single cells. Nat Commun [Internet]. 2017;8(1):14049. Available from: https://doi.org/10.1038/ncomms14049
6. Stuart T, Butler A, Hoffman P, Hafemeister C, Papalexi E, Mauck III WM, et al. Comprehensive Integration of Single-Cell Data. Cell [Internet]. 2019 Jun 13;177(7):1888-1902.e21. Available from: https://doi.org/10.1016/j.cell.2019.05.031
7. Hao Y, Hao S, Andersen-Nissen E, Mauck WM, Zheng S, Butler A, et al. Integrated analysis of multimodal single-cell data. Cell [Internet]. 2021;184(13):3573-3587.e29. Available from: https://www.sciencedirect.com/science/article/pii/S0092867421005833
8. Zilionis R, Engblom C, Pfirschke C, Savova V, Levantini E, Pittet MJ, et al. Single-Cell Transcriptomics of Human and Mouse Lung Cancers Reveals Conserved Myeloid Populations across Individuals and Species Resource Single-Cell Transcriptomics of Human and Mouse Lung Cancers Reveals Conserved Myeloid Populations across Individuals and Species. Immunity [Internet]. 2019;50(5):1317-1334.e10. Available from: https://doi.org/10.1016/j.immuni.2019.03.009
9. Alquicira-Hernandez J, Powell JE. Nebulosa recovers single-cell gene expression signals by kernel density estimation. Bioinformatics [Internet]. 2021 Aug 15;37(16):2485–7. Available from: https://doi.org/10.1093/bioinformatics/btab003

Supplementary Data:

Fig. S1. MIS-A Clinical Images.

Fig. S2. Single-cell RNAseq Clustering Annotation.

Fig. S3. Single cell RNA sequencing (scRNAseq) analysis demonstrates reduction in T cells but increases in B cell and neutrophil populations.

Fig. S4. MIS-A Neutrophil Hematopathology.

Fig. S5. Expanded Data: Immature, B-cell like, and IFN neutrophil subpopulations emerge in MIS-A.

Fig. S6. Neutrophil scRNAseq annotation compared to published critical acute COVID-19 neutrophils subgroups.

Fig. S7. MIS-A Lymphocyte Hematopathology.

Fig S8. MIS-A is associated with emergence of unique plasmablast subsets.

Fig. S9. Expanded Data: Mitochondrial-high, interferon stimulated gene (IFN), and proliferating subpopulations are unique plasmablast subsets in MIS-A.

Fig S10. Overview of B cell and PB subsets by scRNAseq.

Fig. S11. Increased IgA and SARS-CoV2 reactive antibody levels in MIS-A.

Fig. S12. Autoantibody Binding to Human Lung Microvascular Endothelial Cells.

Fig. S13. Rapid induction of microvascular thrombosis in MIS-A.

Fig. S14. High Resolution Images of Microvascular Fibrin

Table S1. Case Clinical Laboratory Values.

Table S2. Case and Controls for Investigational Analysis.

Movie S1. MIS-A Plasma Causes Microvascular Thrombosis.

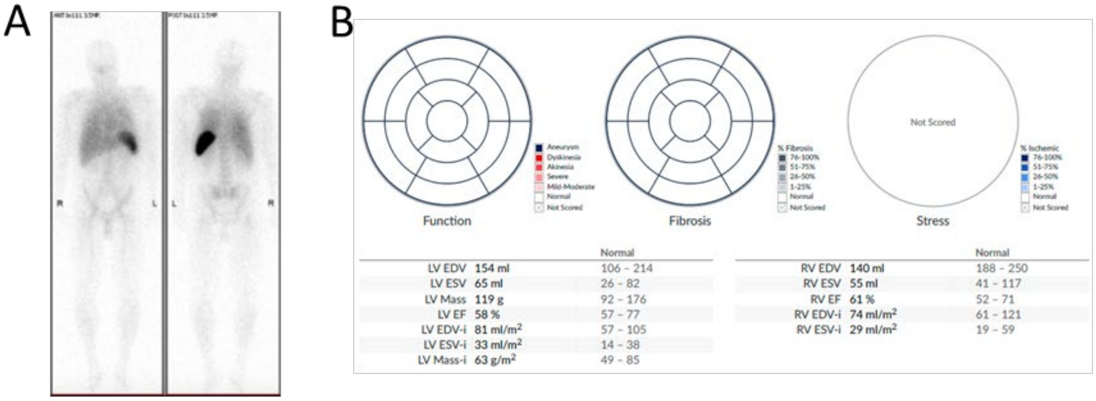

Figure S1 – MIS-A Clinical Images. A) White Blood Cell (WBC) scan whole body image 3.5 hours post injection of indium-111-labeled white blood cells showing what was interpreted by the radiologist to be physiological uptake by the spleen. **B)** Cardiac MRI report just prior to discharge after cardiac function recovery. Cardiac MRI was unable to be obtained acutely due to patients initial SARS-CoV2 PCR positivity (unit infectious diseases precautions) and critical illness (difficulty with transport while on vasopressors).

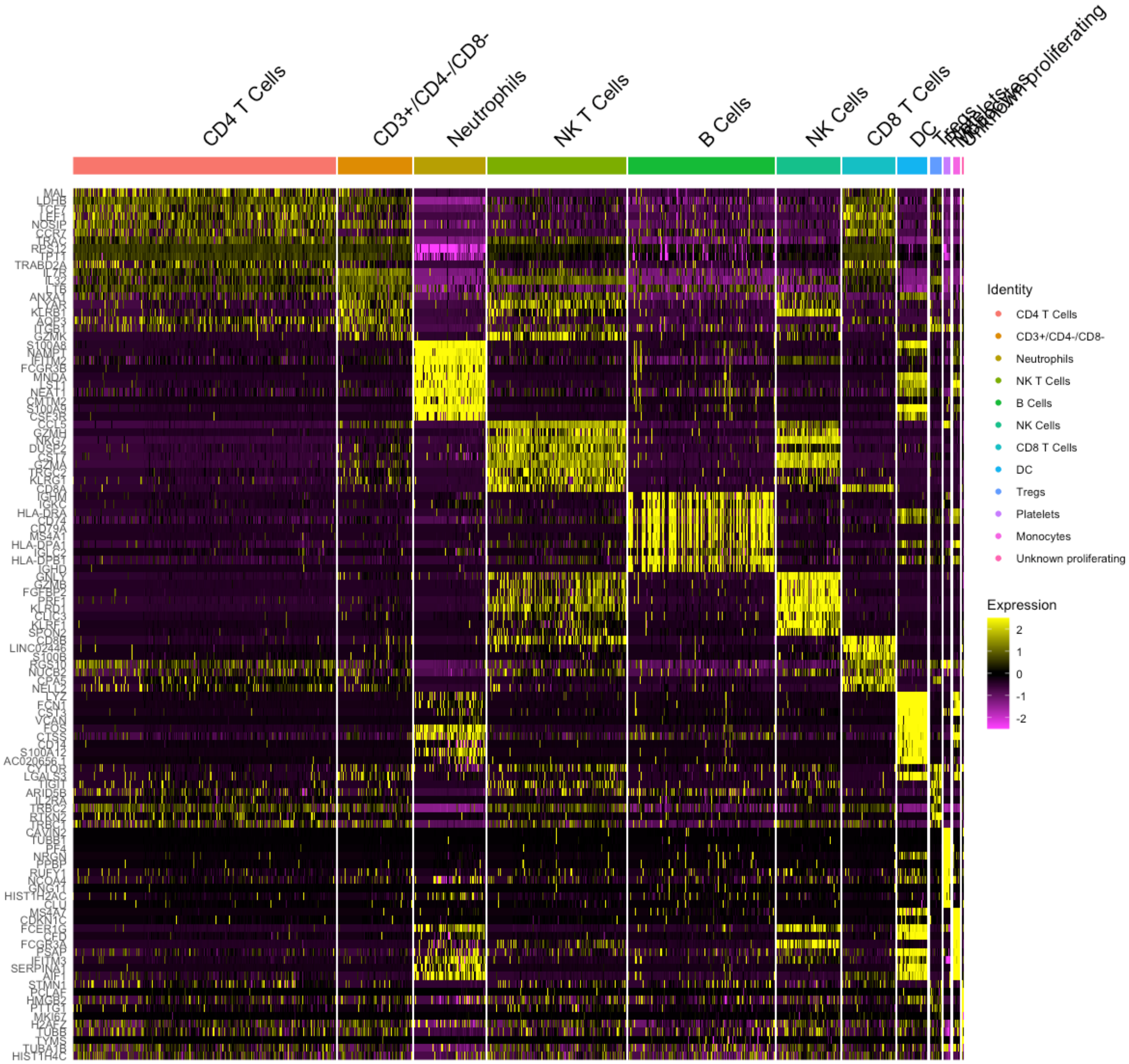

Figure S2 – *Single-cell RNAseq Clustering Annotation.* Immune subtype identification from all patient scRNAseq data using Azimuth annotation. Key cell type specific markers are shown.

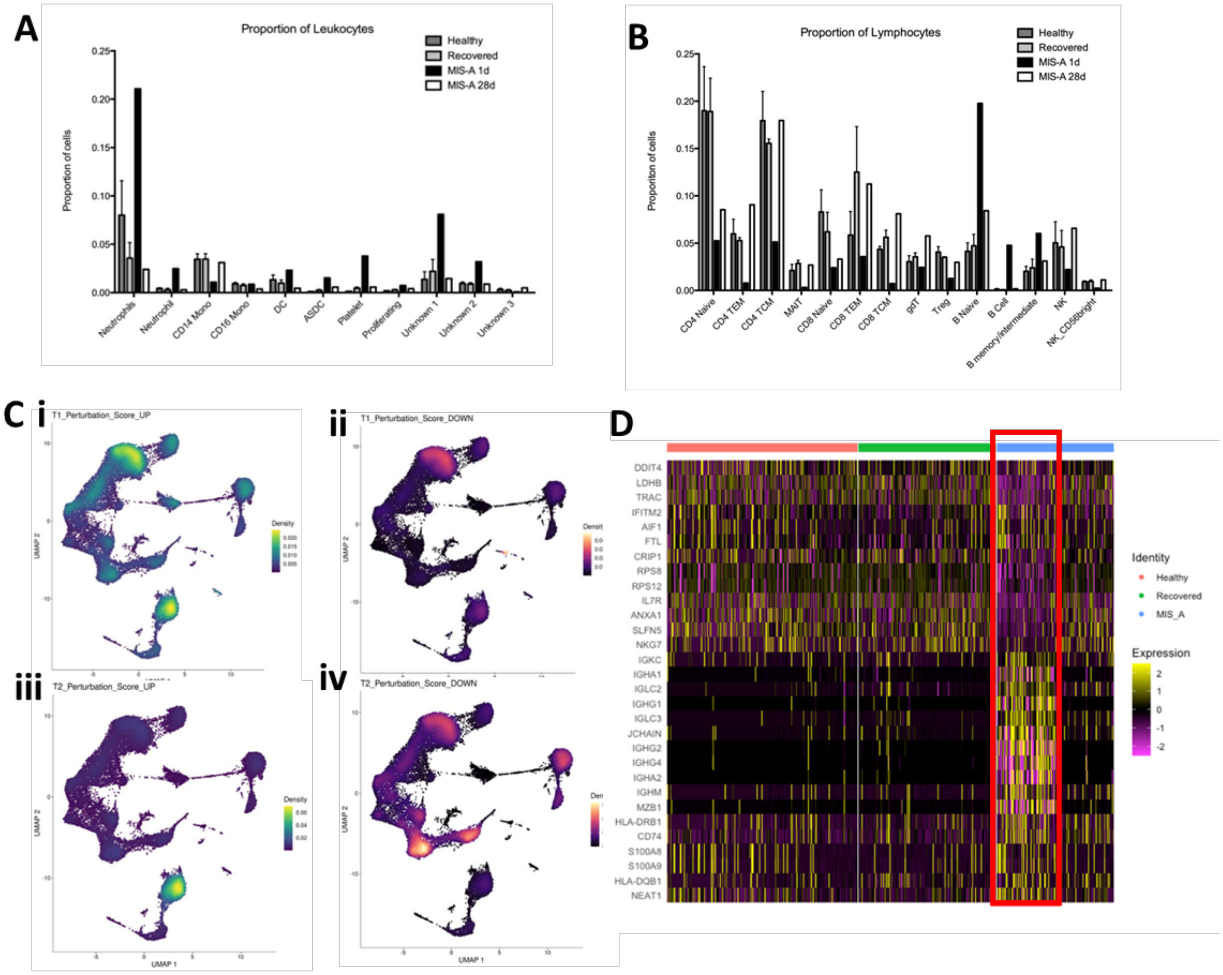

Figure S3 – *Single cell RNA sequencing (scRNAseq) analysis demonstrates reduction in T cells but increases in B cell and neutrophil populations in MIS-A*. A-B) Quantitation of lymphocyte (**A**) and non-lymphocyte (**B**) subpopulations from Figure 1D of main manuscript. **C**) Perturbation scores demonstrating overall transcriptional changes within immune cell populations in acute MIS-A (T1) and recovery MIS-A (T2) shows marked changes in T cell, B cell and Neutrophils acutely with persistent alterations in neutrophils following partial recovery. **D**) Top 25 differentially expressed genes in all immune cells grouped by clinical subgroups show MIS-A, and particularly acute MIS-A (red box), shows marked changes in gene expression including those associated with B cells and neutrophils.

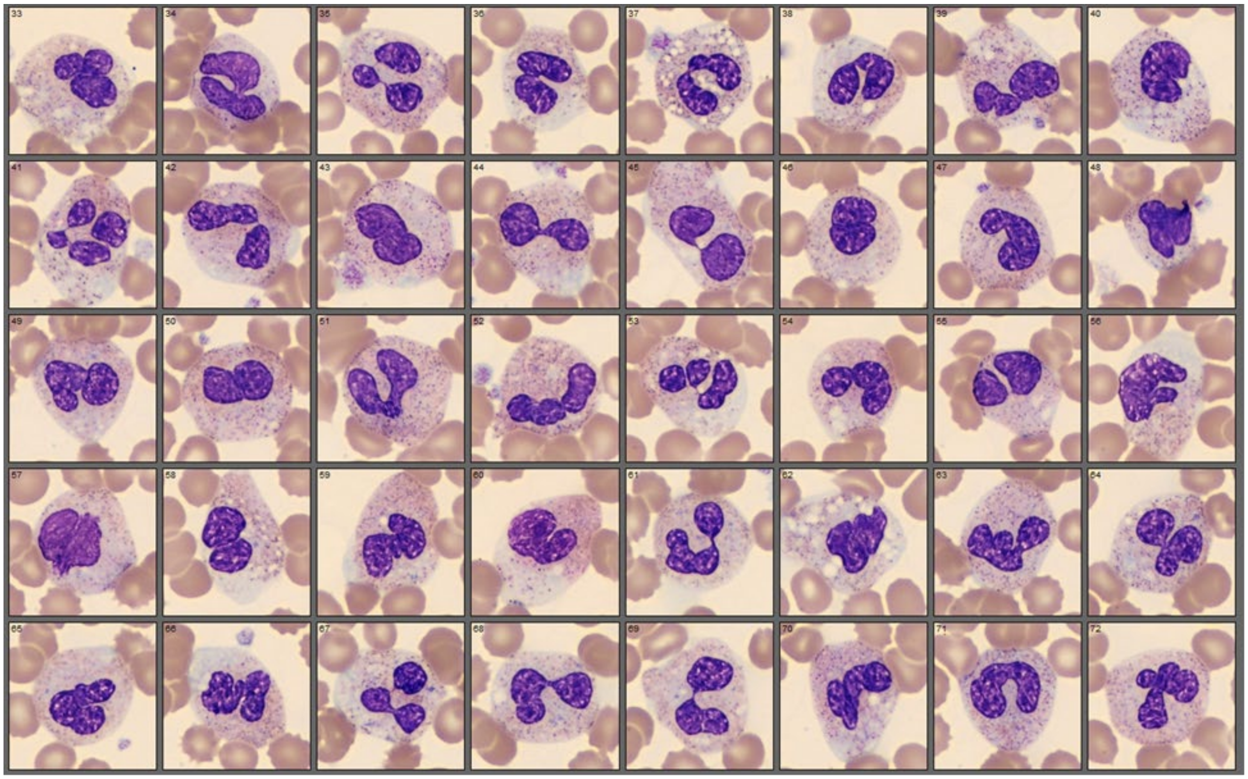

Figure S4 – *MIS-A Neutrophil Hematopathology*. Representative neutrophil images from Cellavision™ analysis of peripheral blood smears collected prospectively and acutely during MIS-A patient hospital stay.

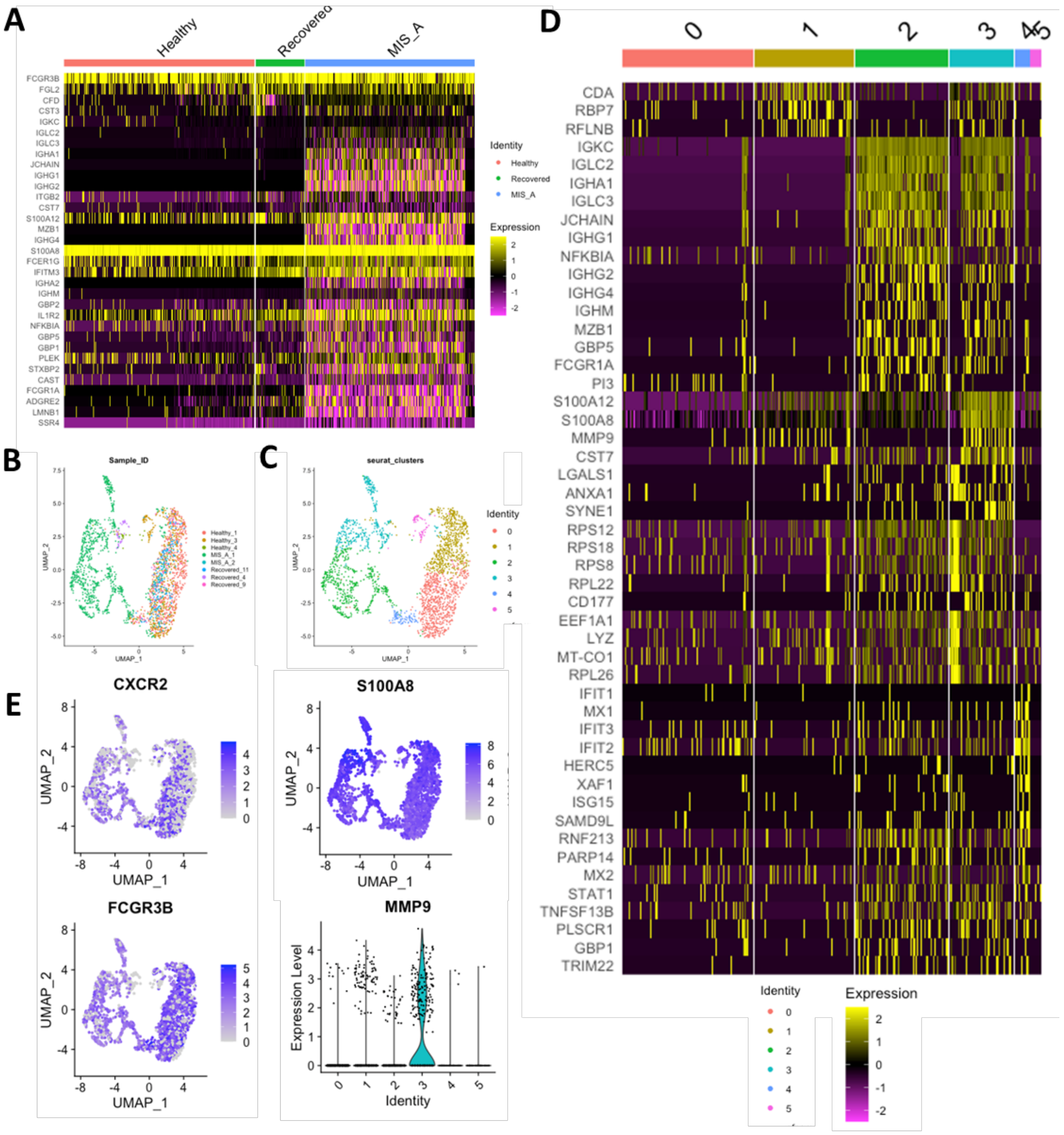

Figure S5 – *Immature, B-cell like, and IFN neutrophil subpopulations emerge in MIS-A*. **A**) Top 25 differentially expressed genes in neutrophils demonstrates marked changes in acute MIS-A. **B**) Unbiased clustering of all patient neutrophils identifies increased and distinct clusters in acute MIS-A. **C**) Six neutrophil subpopulations are identified with subgroups 0 and 1 mainly from healthy donors, subgroup 2-4 from MIS-A, and subgroup 5 largely from a single healthy donor. **D**) Top differentially expressed genes for each cluster from panel B showing subgroups 0 and 1 largely overlapping from healthy donors, subgroup 2 containing high B-cell signatures (Igs, MZB1, Jchain), subgroup 3 consisting of B-cell signatures plus largely LYZ and MMP9 immature populations, subgroup 4 high in interferon IFN-stimulated genes (ISG15, IFIT2,IFIT3), and cluster 5 representing a low transcript neutrophil population. **E**) Classic neutrophil markers CXCR2, S100A8, and FCGR3B. Immature neutrophil marker MMP9 associated with subgroup 3.

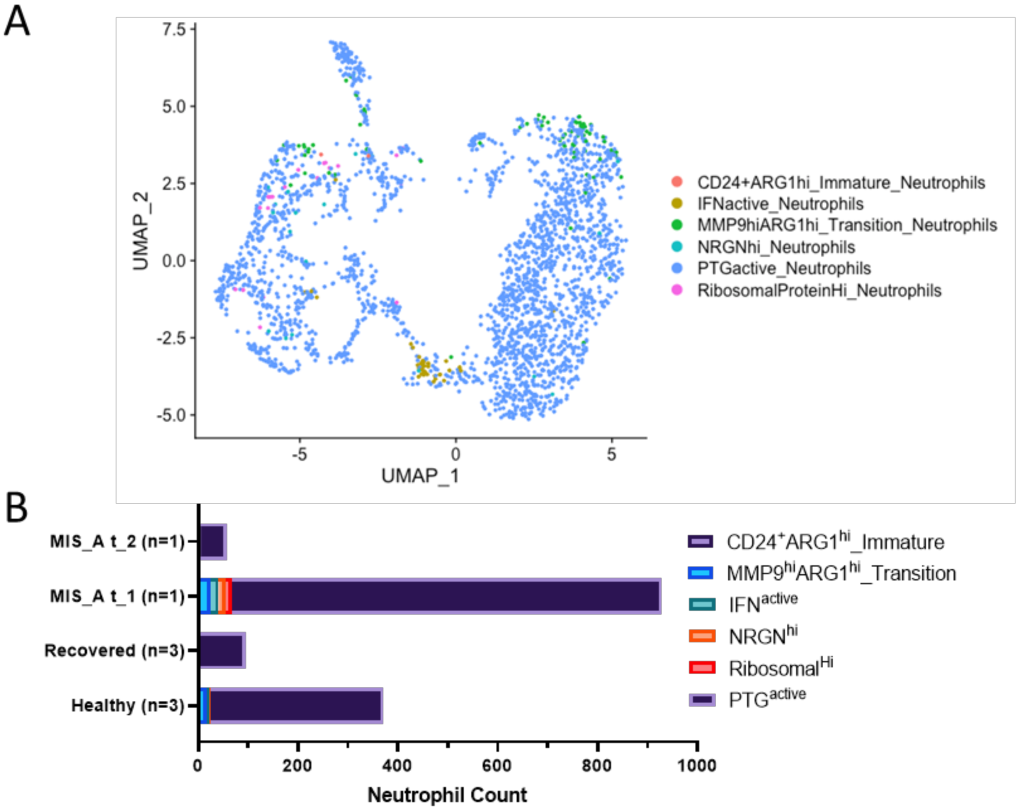

Figure S6 – *Neutrophil scRNAseq annotation compared to published critical acute COVID-19 neutrophils subgroups.* Neutrophil annotation using recently described neutrophil populations identified in critically ill COVID-19 and bacterial acute respiratory distress syndrome (1). **A)** UMAP clustering shown neutrophil annotations. **B)** Average absolute number of neutrophils identified in each scRNAseq sample analyzed in this study.

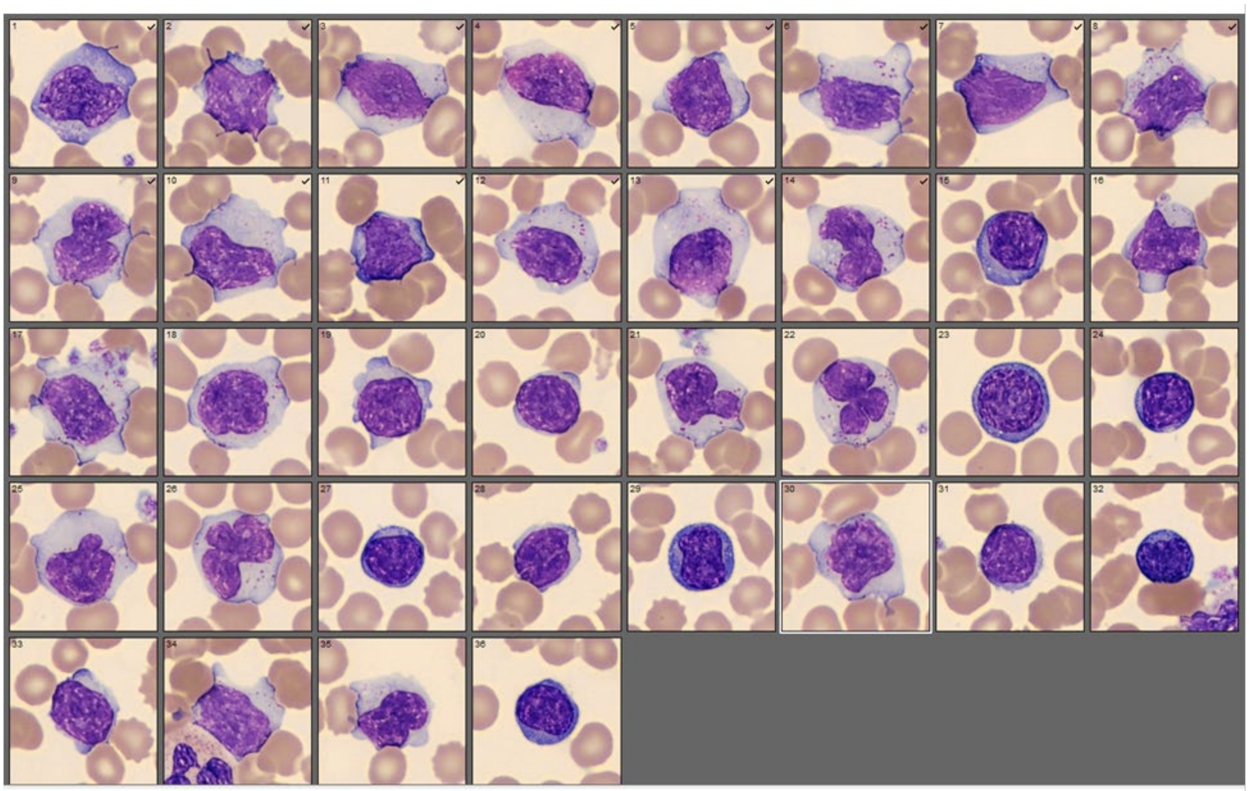

Figure S7 – *MIS-A Lymphocyte Hematopathology*. Representative lymphocyte images from Cellavision™ analysis of peripheral blood smears collected prospectively and acutely during patients stay.

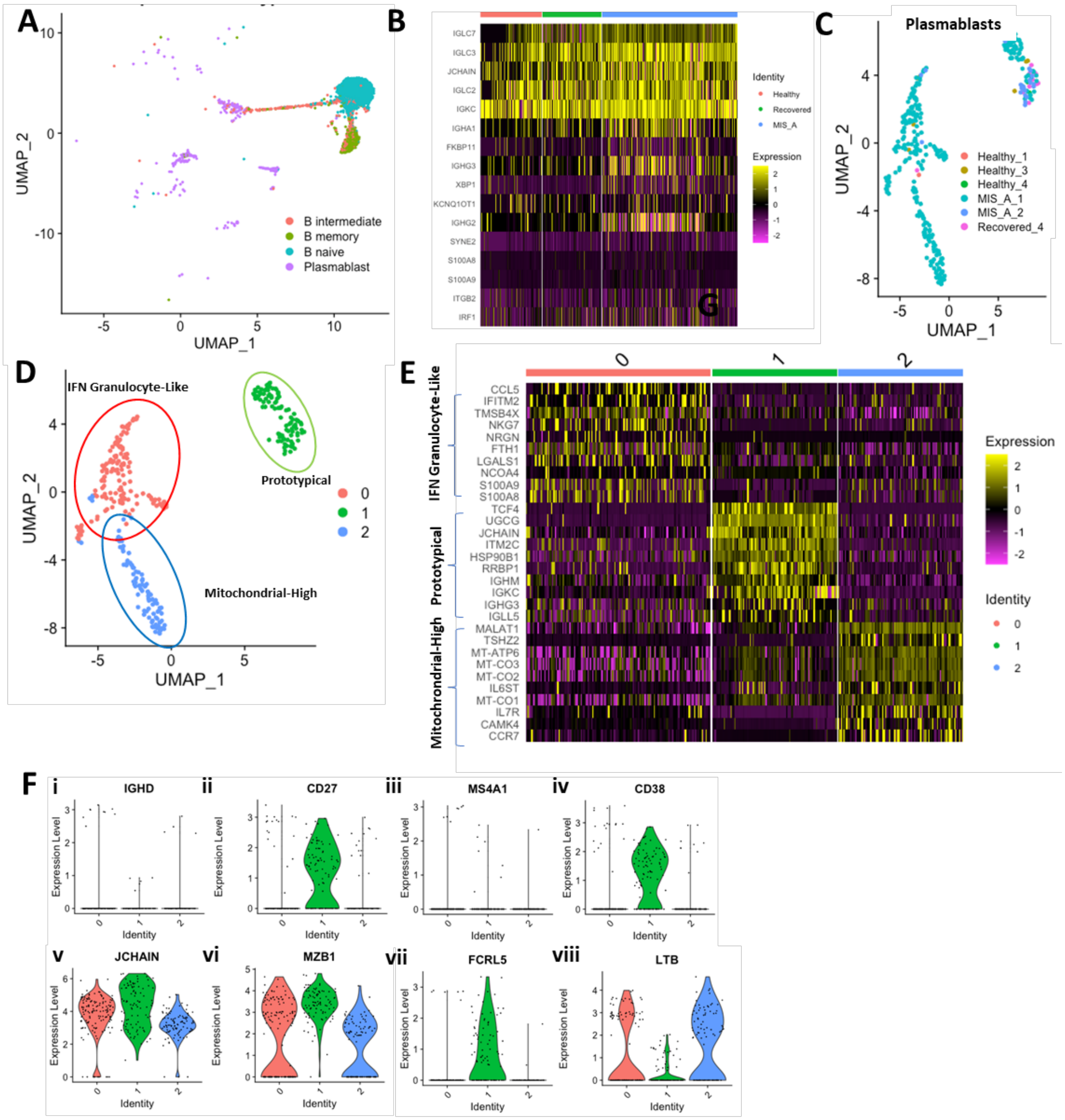

Figure S8 –*MIS-A is associated with emergence of unique plasmablast subsets.* **A**) B-cell clustering using Azimuth reference database showing unknown but unique populations plasmablasts (purple). **B**) Top differentially expressed genes in clinical subgroups by scRNAseq. **C**) Unsupervised clustering of Plasmablasts shows increased plasmablasts in MIS-A. (**D**) Three unique plasmablast subpopulations identified using standard . **E**) Differential gene expression of plasmablast subpopulations identifies novel markers. **F**) Plasmablast subpopulations expressing common B cell markers (i-viii). **Note:** all PB analysis here includes only low (<15%) mitochondrial RNA content cells which is considered the standard cut-off for ‘live’ cells using scRNAseq analysis.

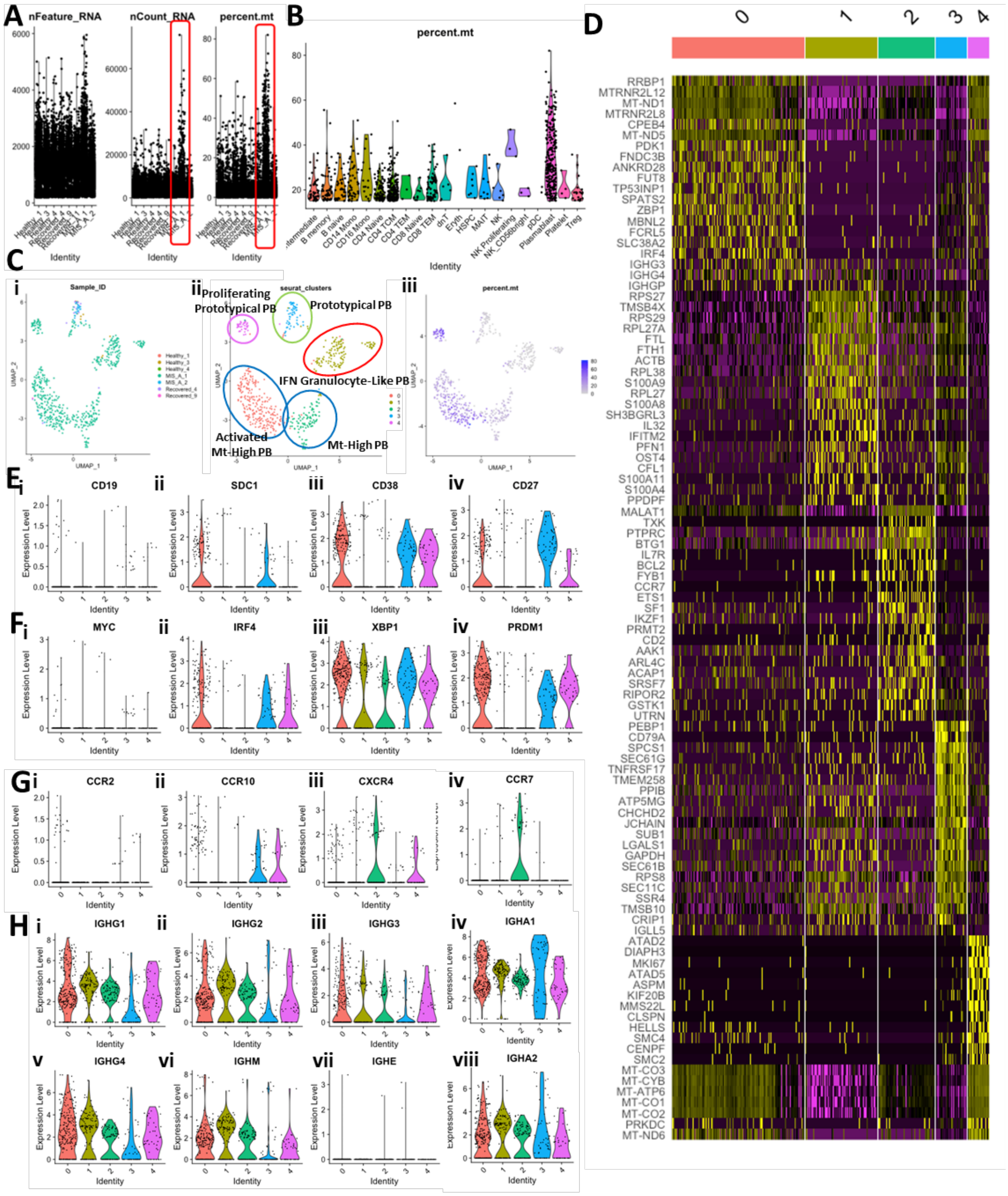

Figure S9 – *Mitochondrial-high, interferon stimulated gene (IFN), and proliferating subpopulations are unique Plasmablast subsets in MIS-A*. A) scRNAseq feature (**i**), nRead (**ii**), and high mitochondrial content (**iii**) is increased in MIS-A. **B)** Plasmablasts are the dominant source of high mitochondrial cells. **C)** Unsupervised clustering of plasmablast populations including high-mitochondrial read (>15%) cells shows elevated counts in MIS-A (**i**) with unique plasmablast subpopulations (**ii**) separated by mitochondrial read levels (**iii**). **D)** Top differentially expressed genes define 5 subpopulations of plasmablasts. **E**) Common B cell surface markers in PBs identified scRNAseq CD19 (**i**), CD138/SDC1 (**ii**), CD38 (**iii**), CD27 (**iv**). **F)** Common B cell transcription factors regulating antibody secreting capacity in PBs identified scRNAseq Myc (**i**), IRF4 (**ii**), XBP1 (**iii**), PDRM1/BLIMP1 (**iv**). **G)** Plasmablast subsets differentially express chemokine receptors known to regulate B cell trafficking (**i-iv**). **H)** Immunoglobulin heavy chain expression by PBs identified scRNAseq (**i-viii**).

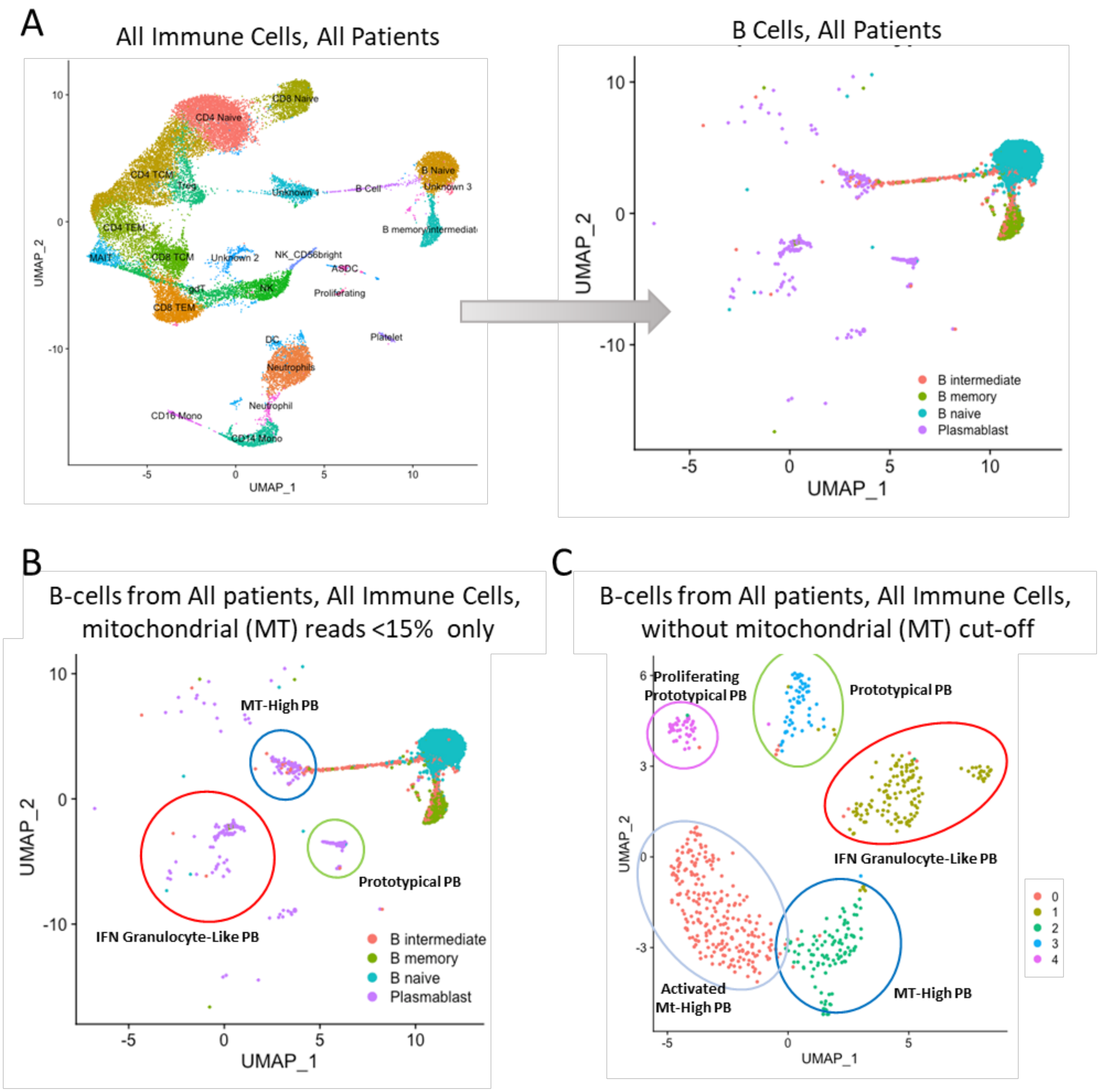

Figure S10 – *Overview of B cell and PB subsets by scRNAseq.* A) Azimuth annotation of all immune cells from all patient groups using initial <15% MT read cut-off (Left) with B cell subpopulations identified in this annotation (Right). **B)** Similar to panel A-Right, Azimuth annotated B cells with three candidate plasmablast subtypes identified. **C)** Unsupervised clustering of all patient plasmablasts including cells with high mitochondrial reads (>15%) previously excluded from analysis due to standard scRNAseq quality control procedures.

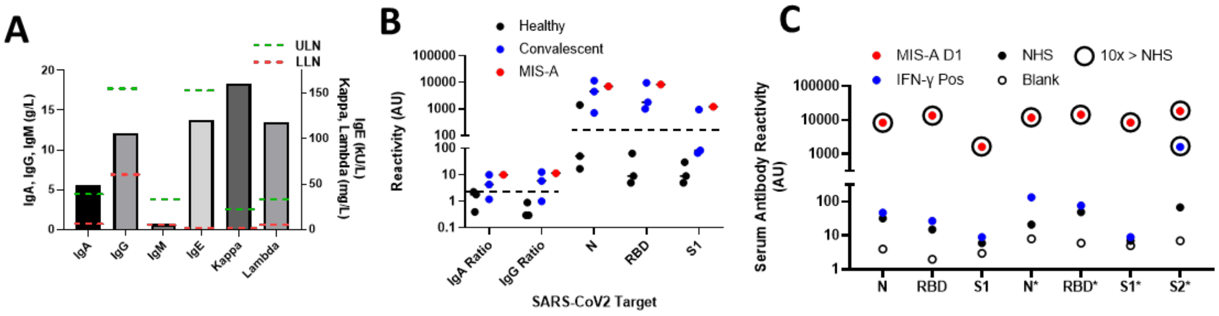

Figure S11 – *Increased IgA and SARS-CoV2 reactive antibody levels in MIS-A.* A) Acute MIS-A immunoglobulin and free Kappa and Lambda light chain levels. B) SARS-CoV2 serology in acute MIS-A versus healthy and convalescent controls. C) Serum auto-antibody reactivity from MIS-A D1 versus known IFN-γ positive patient, and normal human serum (NHS). Values >10x NHS are circled. ULN = upper limit of normal, LLN = lower limit of normal using validated central laboratory cut-offs. AU = arbitrary units.

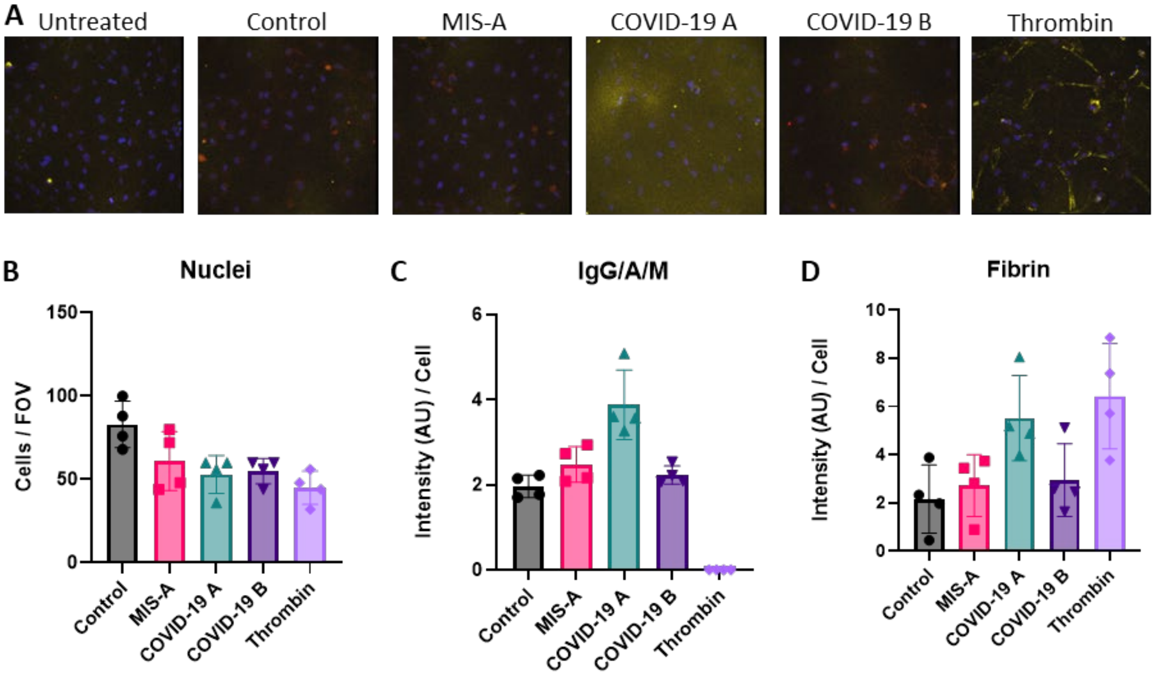

Figure S12 – *MIS-A and acute COVID-19 autoantibody binding to human lung microvascular endothelial cells.* A) Primary human lung microvascular endothelial cells (HLMEC) untreated or treated for 30 minutes with healthy, MIS-A, or acute critical COVID-19 EDTA plasma diluted 1:10 and spiked with Alexa-546 Fibrinogen (**yellow**). Cells were then fixed and stained for nuclei (DNA, **blue**) and human IgG/A/M (**red**). B) Quantitation of HLMEC cells (nuclei) per FOV using experimental set up in A. C) Quantitation of IgG/A/M intensity (AU) using experimental set up in A. D) Quantitation of fibrin intensity per cell using experimental set up in A.

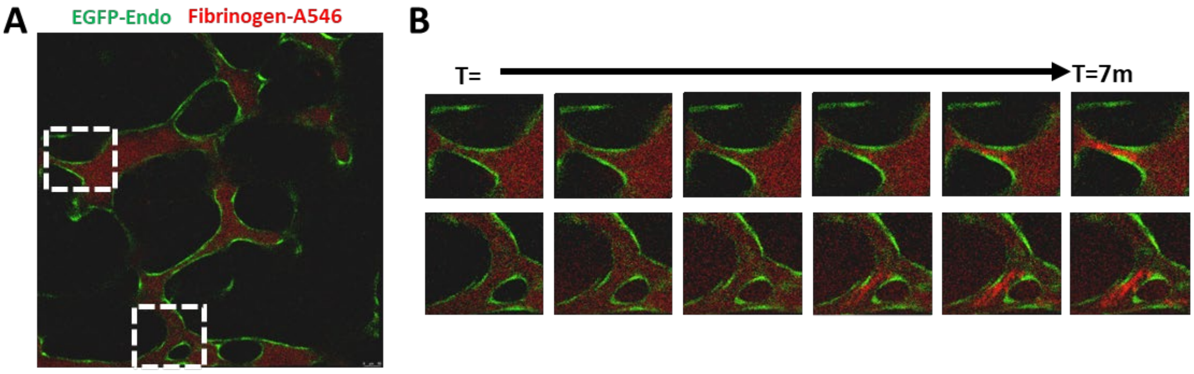

Figure S13 – *Rapid induction of microvascular thrombosis in MIS-A. A*-B) Time lapse series of microvascular obstruction with fibrinogen in presence of MIS-A plasma in circled vessels. Top and bottom boxes in B correspond to top and bottom panel series in C.

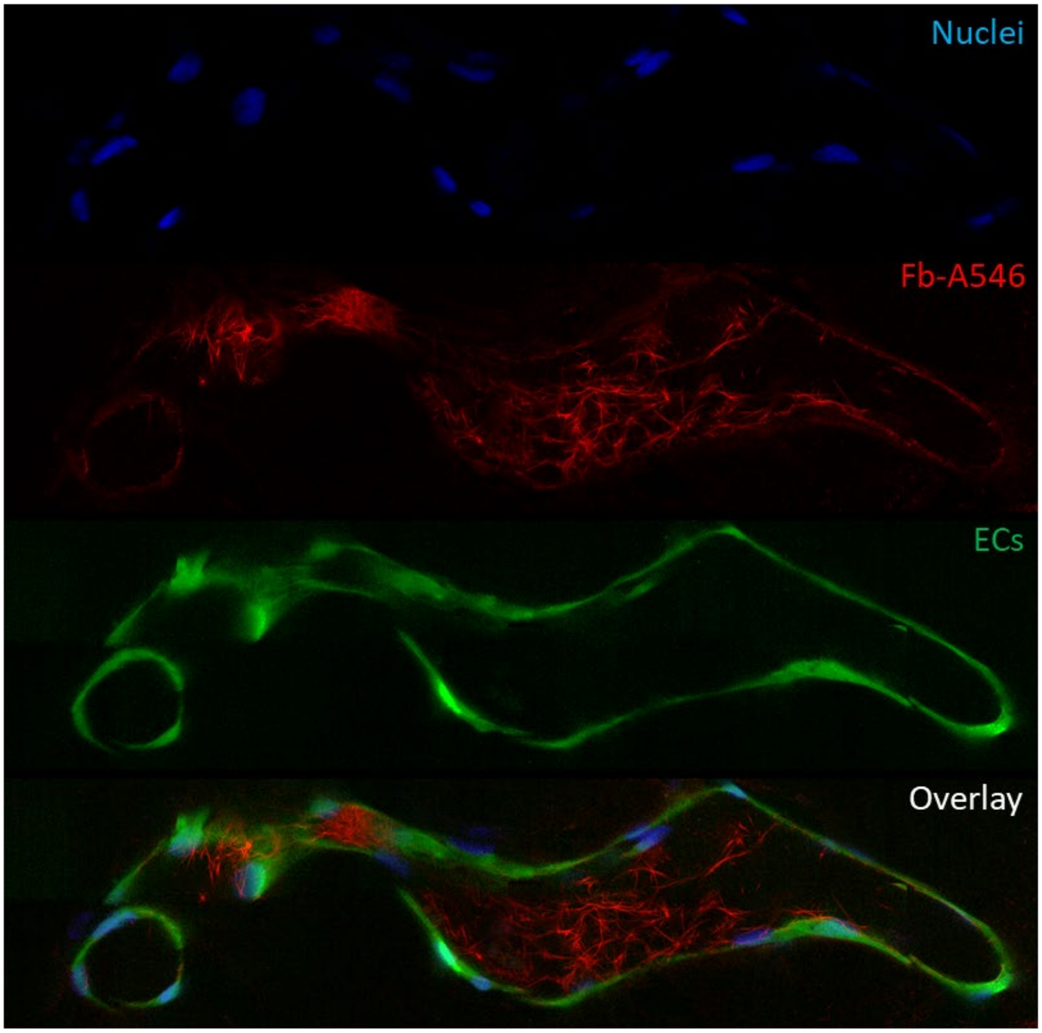

Figure S14 – *High Resolution Images of Microvascular Fibrin.* A) High resolution image of microvascular fibrinogen Alexa-546 accumulation in microvessels in response to MIS-A plasma for 15 minutes from Figure 5.

Table S1- Case Clinical Laboratory Values

Day from Admit	Hgb g/L	WBC *10^9/mL	Monocytes *10^9/mL	Neutrophils *10^9/mL	Imm Gran *10^9/mL	Metamyelo *10^9/mL	Lymphs *10^9/mL	Platelets *10^9/mL
1	136	8	0.6	6.7	0	0	0.5	173
2	123	8.4	0.6	7	0	0	0.6	169
3	121	14.9	0.2	12.9	0.2	0	0.9	173
4	124	20.1	0.7	17.6	0.6	0	0.6	200
5	121	26.2	0.5	23.9	0.2	0	1.1	215
6	116	27.2	0.5	24.1	0.5	0	1.5	227
7	118	25.5	0.8	21.9	0.8	0	1.3	255
8	106	22.4	0.6	18.4	1	0	1	243
9	103	18.2	7	15.1	0.7	0.2	1.1	293
10	97	16.4	0.5	16.4	0.7	0	0.7	313
11	90	13.6	0.5	11	0.3	0	1.1	325
12	87	14.1	0.4	11.4	0.3	0	1.2	338
13	92	12.6	0	10.1	0.1	0	1.7	407
14	100	11.3	0.2	7.6	0.1	0	2.3	418
15	101	12.3	1	8.5	0.1	0	1.7	418
47	118	4.6	0.4	2.3	0	0	1.7	246
Ref Rg	137-180	4.0-11.0	0.0-1.0	2.0-8.0	0	0	0.7-3.5	150-400

	IgA (g/L)	IgG (g/L)	IgM (g/L)	IgE (kU/mL)	Free Kapp	Free Lambda	C3 (g/L)	C4 (g/L)
7	5.54	12.16	0.66	120.7	160.9	118.1	0.42	0.02
47	ND	ND	ND	ND	ND	ND	1.34	0.29
Ref Rg.	0.6-4.2	6.8-18.0	0.4 – 3.0	0.00-160.0	3.3-19.4	5.7-26.3	0.6-1.60	0.1-0.4

	ANA	ENA	DS-DNA	MPO	PR3	GBM	RF
7	Neg	Neg	<1	<0.2	<0.2	<0.2	<10
Ref Rg.	>1:80	Neg	<1	<0.2	<0.2	<0.2	<10

	CRP (mg/L)	Ferritin (µg/L)	D-dimer (mg/L)	Fibrinogen (g/L)	INR (s)	Troponin (ng/L)	NT-pro-BNP (ng/L)
1	202	ND	ND	ND	1.5	503	4025
7	278	1250	6.26	6.5	1.4	200	33, 698
47	0.8	252	0.57	ND	1.1	6	ND
Ref Rg.	0-8	30-500	<0.5	1.6-4.1	0.9-1.1	0-13	0-300

	Sodium (mmol/L)	Albumin (g/L)	ALT (U/L)	GGT (U/L)	Creatinine (µmol/L)	TG (mmol/L)
1	132	ND	40	106	138	1.98
7	129	16	26	64	440	ND
47	136	35	27	41	80	ND
Ref Rg.	133-145	35-45	1-60	11-62	50-120	0-1.7

Table S2 - Case and Controls for Investigational Analysis

Samples	Healthy	Healthy	Healthy	Rocovered	Recovered	Recovered	MIS-A T1	MIS-A T2
Age	44	44	37	35	42	40	38	38
Gender	M	M	F	F	M	M	M	M
COVID-19	No	No	No	Yes	Yes	Yes	Yes	Yes
Primary COVID-19 symptoms:								
Fever				-	+	-	-	
Chills				-	+	-	+	
SOB				-	+	-	-	
Cough				-	+	+	+	
Sore Throat				-	+	+	+	
Fatigue				+	+	+	+	
Loss of smell / taste				+	+	-	-	
COVID-19 Vaccine	No	No	No	No	No	No	No	No
Long COVID*	No	No	No	No	No	No	Yes	Yes
Comorbidities	None	None	None	None	None	None	None	None

* Whether patient would be later considered ‘Long COVID’, defined as >12 weeks of unexplained symptoms from primary infection (symptoms included fatigue, headache, ‘brain fog’, abdominal pain, and gastrointestinal upset)

Movie S1 - MIS-A Plasma Causes Microvascular Thrombosis

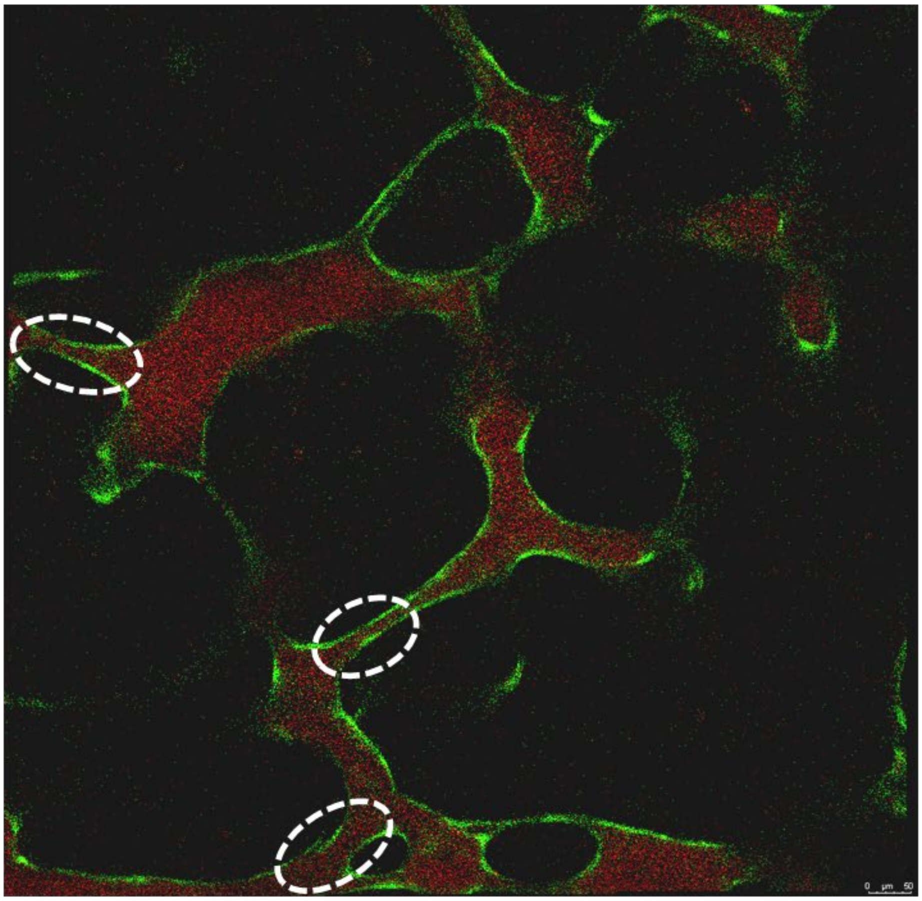

3D Microvascular chips with HUVEC-GFP (green) endothelial cells were perfused for 7 minutes with fresh frozen acute (T1) MIS-A plasma diluted 1:10 in vasculife base media. Live-cell imaging was performed using a Leica Sp8 resonant scanning confocal microscope. Circled areas highlight areas where fibrinogen-Alexa546 (red) accumulates.
